# Impact of Pandemics on Primary Care: Changes in GP Antidepressant Prescriptions and Mental Health Referrals During Lockdowns in England, UK

**DOI:** 10.1192/j.eurpsy.2025.1649

**Published:** 2025-08-26

**Authors:** G. Yu, Y. Fu, E. Y. Tang

**Affiliations:** 1 King’s College London, London; 2 The University of Liverpool, Liverpool; 3 Newcastle University, Newcastle, United Kingdom

## Abstract

**Introduction:**

The COVID-19 pandemic disrupted primary healthcare services, affecting mental health support and antidepressant prescriptions in England. This study examines shifts in GP referrals and antidepressant prescribing patterns during different phases of lockdown.

**Objectives:**

This research aims to (1) analyze changes in the rates of antidepressant prescriptions across lockdown periods, and (2) investigate how GP referrals to mental health services, including social prescribing, evolved, with a focus on disparities among ethnic groups.

**Methods:**

Using a retrospective design, we analyzed electronic health record data from GP practices in North England, covering March 2018 to June 2023. We employed a two-level negative binomial-logit hurdle model for antidepressant prescriptions and a multinomial logit regression model for referral types.

**Results:**

Antidepressant initiation decreased during lockdowns, while ongoing prescriptions showed minor increases. GP referrals to social prescribing rose significantly, especially among ethnic minorities who also had fewer medical treatments. Lockdown phases influenced referral patterns, with reductions in secondary care referrals and growth in community-based support.

**Conclusions:**

The study highlights a shift towards social prescribing amid the mental health strains of the pandemic, suggesting its role in a social model of health. Ethnic disparities in mental health care access emphasize the need for culturally inclusive, non-clinical mental health support.

**Disclosure of Interest:**

None Declared

